# A phase 1/1b, open-label, dose-escalation study of PD-1 inhibitor, cetrelimab alone and in combination with FGFR inhibitor, erdafitinib in Japanese patients with advanced solid tumors

**DOI:** 10.1007/s10637-024-01433-3

**Published:** 2024-06-04

**Authors:** Noboru Yamamoto, Yasutoshi Kuboki, Kenichi Harano, Takafumi Koyama, Shunsuke Kondo, Akiko Hagiwara, Noriko Suzuki, Ei Fujikawa, Kiichiro Toyoizumi, Mayumi Mukai, Toshihiko Doi

**Affiliations:** 1 Department of Experimental Therapeutics, National Cancer Center Hospital, Tokyo, Japan; 2 Department of Experimental Therapeutics, National Cancer Center Hospital East, Chiba, Japan; 3 Research and Development Division, Janssen Pharmaceutical K.K., Tokyo, Japan

**Keywords:** FGFR inhibitor, Immune checkpoint inhibitors, Monoclonal antibody, Small molecule inhibitor, Targeted cancer therapy

## Abstract

Immune checkpoint inhibitors are the leading approaches in tumor immunotherapy. The aim of the study was to establish recommended phase 2 doses (RP2Ds) of intravenous cetrelimab, a checkpoint inhibitor, alone and with oral erdafitinib in Japanese patients with advanced solid tumors. This open-label, non-randomized, dose-escalation phase 1/1b study enrolled adults with advanced solid tumors who were ineligible for standard therapy. Study was conducted in two parts: phase 1a assessed cetrelimab at three dosing levels (80 mg every 2 weeks [Q2W], 240 mg Q2W, and 480 mg Q4W); phase 1b assessed cetrelimab+erdafitinib at two dosing levels (240 mg Q2W + 6 mg once daily [QD] and 240 mg Q2W + 8 mg QD). Primary endpoint was frequency and severity of dose-limiting toxicities (DLTs) of cetrelimab ± erdafitinib. In total 22 patients (phase 1a, *n* = 9; phase 1b, *n* = 13) were enrolled. Median duration of follow-up was 8.64 months in phase 1a and 2.33 months in phase 1b. In phase 1a, DLTs weren’t reported while in phase 1b, 1 patient who received 240 mg cetrelimab + 6 mg erdafitinib reported Stevens-Johnson syndrome (grade 3, immune-related). Overall, 88.9% patients in phase 1a (grade ≥ 3: 44.4%) and 100.0% in phase 1b (grade ≥ 3: 53.8%) experienced ≥ 1 treatment-related adverse events (TEAEs); 33.3% in phase 1a and 38.5% in phase 1b reported serious TEAEs, of which 11.1% patients in phase 1a and 15.4% in phase 1b had TEAEs which led to treatment discontinuation. Cetrelimab alone and in combination with erdafitinib showed manageable safety in Japanese patients with advanced solid tumors. RP2Ds were determined as 480 mg cetrelimab Q4W for monotherapy, and cetrelimab 240 mg Q2W + erdafitinib 8 mg QD for combination therapy.

**Supplementary Information:**

The online version contains supplementary material available at 10.1007/s10637-024-01433-3.

## Introduction

Monoclonal antibodies make up most of the immune checkpoint inhibitors and are designed to precisely target cancer cells by blocking the checkpoint proteins, programmed cell death protein-1 (PD-1), and PD-ligand (L)1. The immune checkpoint inhibitors prevent PD-L1 on tumor cells from binding to PD-1 on T-cells enabling them to destroy tumor cells [1]. In contrast, small molecule inhibitors potentially offer a prospective route of targeted cancer therapy by interfering with the proteins and genes involved in cancer cell growth [2]. Combination therapies, integrating monoclonal antibodies with small molecules for targeting the cancer cells, have been investigated in both preclinical and clinical trials [3–7].

Cetrelimab, a checkpoint inhibitor, is a fully human immunoglobulin (Ig)G4 kappa monoclonal antibody targeting PD-1 on the T-lymphocytes. It is currently under development as mono and combination therapy. The first-in-human global phase 1/2 clinical study in patients with advanced/refractory solid tumors reported the safety and preliminary efficacy of cetrelimab monotherapy (intravenous) while determining the recommended phase 2 doses (RP2Ds) as 240 mg every 2 weeks (Q2W) or 480 mg Q4W [8]. Cetrelimab is also being investigated in combination regimens for treating cancers [9–11].

Erdafitinib is an oral small molecule pan inhibitor of fibroblast growth factor receptor (FGFR) tyrosine kinase which may be involved in the proliferation, differentiation, and migration of cancer cells. It has received accelerated approval from the US Food and Drug Administration (FDA) for patients with metastatic or locally advanced urothelial cancer, with susceptible *FGFR3* or *FGFR2* genetic alterations [12]. Previous phase 1 and 2 studies of erdafitinib as monotherapy investigated safety and preliminary efficacy in solid tumors [13–16] and in the phase 3 study (THOR) erdafitinib prolonged overall survival significantly in patients with advanced or metastatic urothelial carcinoma with *FGFR* aberrations, compared to standard chemotherapy [17]. The efficacy results of the phase 2 study (NORSE) which evaluated erdafitinib plus cetrelimab in metastatic/advanced urothelial carcinoma favored combination therapy over erdafitinib alone [7].

The current study was aimed to determine the RP2Ds of cetrelimab alone and in combination with erdafitinib in Japanese patients with advanced solid tumors. Additionally, the safety, pharmacokinetics (PK), immunogenicity, pharmacodynamics, and antitumor activity of cetrelimab as monotherapy and in combination with erdafitinib were assessed.

## Methods

### Study design

This open-label, non-randomized, phase 1/1b study was conducted in two parts: phase 1a (dose-escalation study of cetrelimab) and phase 1b (dose-escalation study of cetrelimab + erdafitinib). Institutional Review Board at all participating institutions approved the study protocol and associated study documents. Good Clinical Practice guidelines by the International Council for Harmonization and all relevant regulatory requirements were followed during the study. All patients gave their informed consent before enrollment.

### Patients

Patients aged ≥ 20 years with a confirmed diagnosis of advanced/refractory, metastatic, or unresectable solid tumors who had previously received or were ineligible for standard therapy, with Eastern Cooperative Oncology Group (ECOG) performance status 0 or 1, were included. Minimum of two patients who had confirmed positive *FGFR* aberrations confirmed either locally or centrally were planned to be included in the cetrelimab 240 mg Q2W + erdafitinib 8 mg QD cohort of phase 1b part of the study. Patients with uncontrollable disease or active infection (requiring antibiotics via intravenous route); those who had prior treatment with an anti-PD-1 or anti-PD-L1 or anti-PD-L2 antibodies within 30 days of first study drug administration; ongoing grade **≥** 2 immunotherapy-related toxicity; grade **≥** 3 toxicity effects from previous treatment with immunotherapy; history of concurrent interstitial lung disease; an autoimmune disease which requires systemic steroid or immunosuppressive agents; patients who had antineoplastic therapy, radiotherapy, or treatment with an investigational study drug 14 days prior to initiation of the study; who had autoimmune diseases were excluded.

### Treatment

Phase 1a part of the study evaluated three dosing levels of cetrelimab as intravenous infusion (80 mg Q2W, 240 mg Q2W, and 480 mg Q4W) in three dose-escalating cohorts. While in phase 1b part, cetrelimab (RP2D established in phase 1a part) in combination with two dosing levels of erdafitinib as oral film-coated tablets (cetrelimab 240 mg Q2W + erdafitinib 6 mg once daily [QD], and cetrelimab 240 mg Q2W + erdafitinib 8 mg QD) were given in two combination dose cohorts. Dosing interval was amended based on emerging data.

### Study endpoints and assessments

The primary endpoint was to assess the frequency and severity of dose-limiting toxicities (DLTs) of cetrelimab alone (phase 1a), and when combined with erdafitinib (phase 1b). DLTs included any non-hematological toxicities of grade ≥ 3 (for grade 3 only, except asthenia, anorexia, fever, constipation, nausea [lasting for ≤ 7 days], fatigue [improves to grade ≤ 2 in ≤ 7 days], vomiting and diarrhea [resolves in ≤ 3 days with standard care], laboratory abnormalities not requiring hospitalization and deemed not clinically significant per investigator, tumor flare [local pain, irritation, or rash localized at known or suspected tumor areas, improves to grade ≤ 2 in ≤ 7 days], raised aspartate aminotransferase/alanine aminotransferase [AST/ALT] levels [lasting for < 7 days], hyperphosphatemia [applicable to erdafitinib combination], increased AST/ALT levels that meet Hy’s law criteria). Hematological toxicities (including grade 4 neutropenia lasting > 7 days, febrile neutropenia, thrombocytopenia [requires platelet transfusion, grade 3 with clinically significant bleeding or grade 4], grade 4 anemia, any grade 5 toxicity) were included.

Secondary endpoints were frequency and severity of adverse events (AEs) and immune-related AEs (irAEs) (phase 1a and phase 1b); determination of serum cetrelimab PK parameters in both parts of the study and erdafitinib in phase 1b part; and the incidence of anti-cetrelimab antibodies (phase 1a and phase 1b). Efficacy of cetrelimab monotherapy and in combination with erdafitinib was also evaluated as an exploratory assessment. RP2D was established at the dose where < 25% probability that the estimated DLT rate is in the excessive toxicity interval based on escalation with overdose control principle, along with overall safety, PK, pharmacodynamics, and efficacy results.

#### Safety

Safety was assessed by monitoring the incidence and severity of AEs, with clinical lab tests, vital signs, and 12-lead electrocardiogram (ECG). DLTs were evaluated during the DLT-evaluation period (4 weeks [for Q2W and Q4W dosing regimen] from the start of the first study drug administration). The severity of AEs was graded per the National Cancer Institute Common Terminology Criteria for Adverse Events (NCI-CTCAE), version 4.03.

#### Pharmacokinetics

Blood samples were collected to evaluate the PK of cetrelimab (both phase 1a and 1b), and erdafitinib (phase 1b). The collected samples on days 1, 2, 4, 8, and 15 of cycle 1 (pre-dose and end of infusion [EOI]); on day 1 of cycles 2 and 3; days 1, 2, 4, 8, and 15 of cycle 4 (pre-dose and EOI); and on day 1 (pre-dose and EOI) of subsequent cycles; and at the final visit.

#### Immunogenicity

Immunogenicity was assessed by evaluating anti-cetrelimab antibodies. The incidence of these antibodies was determined in the patients who had ≥ 1 dose of cetrelimab and appropriate samples, were assessed in both phase 1a and 1b parts of the study.

#### Pharmacodynamics

The PD-1 receptor occupancy by cetrelimab on circulating CD3+, CD3+/CD4+, and CD3+/CD8 + cells was assessed by flow cytometry, in both phase 1a and 1b parts of the study within 2 h post-dose at all intravenous dose levels.

#### Efficacy

Efficacy assessments were evaluated per Response Evaluation Criteria in Solid Tumors (RECIST) version 1.1., and included best overall responses (BORs), objective response rate (ORR), duration of response (DOR), and progression-free survival (PFS).

### Statistical analysis

RP2D identification was guided based on the probability of DLTs by the Bayesian logistic regression model and escalation with the overdose control principle. No formal statistical hypothesis was established. Safety, PK, immunogenicity, pharmacodynamics, and efficacy data were summarized descriptively. DLTs were evaluated in the DLT evaluable analysis set (patients who had ≥ 1 dose of the study drug and those who didn’t discontinue the study during the DLT-evaluation period). Safety and efficacy were assessed in the all-treated analysis set (patients who had ≥ 1 dose of the study drug). PK was evaluated in the PK analysis set (patients who received ≥ 1 dose of the study drug and had ≥ 1 evaluable study drug concentration data) and immunogenicity was determined in the immunogenicity analysis set (patients who had ≥ 1 dose of the study drug and had quantifiable antibodies of study drug in serum samples). Efficacy endpoints in the response evaluable analysis set (patients who received ≥ 1 dose of the study drug and had ≥ 1 post treatment disease assessment) were analyzed according to RECIST v1.1. PFS and DOR were estimated using the Kaplan-Meier method.

## Results

A total of 22 patients (phase 1a [*n* = 9]; phase 1b [*n* = 13]) were enrolled from 2 sites in Japan. Phase 1a part of the study was carried out between September 2018 and February 2021 and phase 1b part between August 2019 and July 2022. In phase 1a part, 9 patients were enrolled and divided into 3 dose-escalation cohorts (3 patients in each cohort) to receive cetrelimab monotherapy. All patients (9/9 [100%]) in phase 1a part completed DLT-evaluation period; 8/9 (88.9%) patients discontinued the treatment and terminated from the study due to progressive disease (PD). In phase 1b part, 13 enrolled patients were assigned to 2 dose-escalation cohorts to receive combination therapy. Two patients with confirmed positive *FGFR* aberrations (*FGFR3* [R248C] and *FGFR2* [FOXP1 fusion]) were enrolled in the cetrelimab 240 mg Q2W + erdafitinib 8 mg QD cohort. Of 13 patients, 12 (92.3%) completed the DLT-evaluation period. Treatment was discontinued by 10/13 (76.9%) patients which was mostly due to PD (7/10 [70.0%]). Three patients in phase 1b part of the study who died during the study were considered to have completed the study.

### Patient characteristics

In phase 1a part, most patients enrolled were men (7/9 [77.8%]) and median age of patients was 66 years with an ECOG performance status of 0 in 6/9 (66.7%) patients. After identifying RP2D in phase 1a part, phase 1b part was initiated. Half of the enrolled patients were men (7/13 [53.8%]) and median age of patients was 68 years in phase 1b part and most of the patients had an ECOG performance status of 0 (10/13 [76.9%]) (Table 1). At the time of screening, stage IV tumor was diagnosed in 5/9 (55.6%) and 11/13 (84.6%) patients in phase 1a and phase 1b parts of the study, respectively.

**Table 1:** Patient demographics and baseline characteristics (all-treated analysis set)

Characteristics	Phase 1a (cetrelimab)				Phase 1b (cetrelimab + erdafitinib)		
	80 mg Q2W (*n* = 3)	240 mg Q2W (*n* = 3)	480 mg Q4W (*n* = 3)	Total (*n* = 9)	240 mg Q2W + 6 mg QD (*n* = 7)	240 mg Q2W + 8 mg QD (*n* = 6)	Total (*n* = 13)
Age (median), years	48.0	69.0	54.0	66.0	69.0	66.0	68.0
Sex							
Men	3 (100.0)	2 (66.7)	2 (66.7)	7 (77.8)	4 (57.1)	3 (50.0)	7 (53.8)
Women	0 (0.0)	1 (33.3)	1 (33.3)	2 (22.2)	3 (42.9)	3 (50.0)	6 (46.2)
ECOG performance status							
0	2 (66.7)	2 (66.7)	2 (66.7)	6 (66.7)	6 (85.7)	4 (66.7)	10 (76.9)
1	1 (33.3)	1 (33.3)	1 (33.3)	3 (33.3)	1 (14.3)	2 (33.3)	3 (23.1)
Time to first diagnosis of cancer, mean (SD), months	27.0 (29.92)	52.6 (35.32)	32.8 (21.64)	37.5 (28.06)	23.3 (17.79)	33.9 (9.00)	28.2 (14.91)
Primary cancer diagnosis							
Adrenal	0	0	1 (33.3)	1 (11.1)	0	0	0
Biliary tract	0	0	0	0	1 (14.3)	0	1 (7.7)
Bladder	0	0	0	0	0	1 (16.7)	1 (7.7)
Colorectal	1 (33.3)	1 (33.3)	0	2 (22.2)	1 (14.3)	0	1 (7.7)
Esophageal	0	0	1 (33.3)	1 (11.1)	2 (28.6)	0	2 (15.4)
Gastric	0	0	0	0	2 (28.6)	0	2 (15.4)
Lung	0	0	0	0	0	2 (33.3)	2 (15.4)
Malignant peritoneal mesothelioma	1 (33.3)	0	0	1 (11.1)	0	0	0
Others	1 (33.3)	2 (66.7)	1 (33.3)	4 (44.4)	1 (14.3)	3 (50.0)	4 (30.8)
PD-L1 status*							
< 1% expression level	2 (66.6)	3 (100.0)	0	5 (55.6)	4 (57.1)	3 (50.0)	7 (53.8)
≥ 1% and < 50% expression level	1 (33.3)	0	1 (33.3)	2 (22.2)	2 (28.6)	0	2 (15.4)
≥ 50% expression level	0	0	0	0	1 (14.3)	0	1 (7.7)
Indeterminate	0	0	0	0	0	1 (16.7)	1 (7.7)
Prior systemic anticancer therapies, median (range)	4.0 (1–8)	5.0 (2–8)	2.0 (1–4)	4.0 (1–8)	3.0 (1–5)	3.5 (3–8)	3.0 (1–8)

All values are expressed as n (%), unless specified. *Two patients in combination part of the study have missing values. ECOG, Eastern Cooperative Oncology Group; PD-L1, programmed cell death ligand 1; Q2W, every 2 weeks; Q4W, every 4 weeks; QD, once daily; SD, standard deviation

### Treatment exposure

In phase 1a part of the study, the median (range) total duration of therapy with cetrelimab was 7.39 (0.0–21.7) months, and the median (range) duration of follow-up (time interval from the date of first dose to the date of death or last day on the study) was 8.64 (1.1–23.1) months. In phase 1b part, the median (range) duration of combination treatment was 2.33 (0.5–5.3) months and the median (range) duration of follow-up was 3.71 (1.5–5.7) months.

### Safety

In phase 1a part of the study, 8/9 (88.9%) patients experienced ≥ 1 treatment-emergent AEs (TEAEs) (Table 2). The most common TEAEs of grade ≥ 3 include decreased appetite, fulminant type 1diabetes mellitus, hyperuricemia, abnormal hepatic function, increased aspartate aminotransferase, blood alkaline phosphatase and gamma-glutamyl transferase reported in 1/9 (11.1%) patients each. Serious TEAEs were observed in 3/9 (33.3%) patients (1 [11.1%] patient in each cohort), of which 2/9 (22.2%) patients had cetrelimab-related serious TEAEs. Serious TEAEs include decreased appetite, fulminant type 1 diabetes mellitus and abnormal hepatic dysfunction (1/9 [11.1%] patients each, grade ≥ 3). Cetrelimab-related irAEs of special interest were reported in 4/9 (44.4%) patients and the most common was rash (3/9 [33.3%]) (Online Resource 1). No patient in 480 mg cohort had treatment-emergent irAEs, cetrelimab-related serious TEAEs, and grade ≥ 3 AEs.

**Table 2:** Summary of TEAEs (all-treated analysis set)

Characteristics, n (%)	Phase 1a (cetrelimab)				Phase 1b (cetrelimab + erdafitinib)		
	80 mg Q2W (*n* = 3)	240 mg Q2W (*n* = 3)	480 mg Q4W (*n* = 3)	Total (*n* = 9)	240 mg Q2W + 6 mg QD (*n* = 7)	240 mg Q2W + 8 mg QD (*n* = 6)	Total (*n* = 13)
Patients with ≥ 1 TEAE	3 (100.0)	3 (100.0)	2 (66.7)	8 (88.9)	7 (100.0)	6 (100.0)	13 (100.0)
Treatment-related	2 (66.7)	3 (100.0)	1 (33.3)	6 (66.7)	7 (100.0)	6 (100.0)	13 (100.0)
Serious TEAEs	1 (33.3)	1 (33.3)	1 (33.3)	3 (33.3)	3 (42.9)	2 (33.3)	5 (38.5)
Treatment-related	1 (33.3)	1 (33.3)	0	2 (22.2)	1 (14.3)	1 (16.7)	2 (15.4)
TEAEs of grade ≥ 3	1 (33.3)	2 (66.7)	1 (33.3)	4 (44.4)	4 (57.1)	3 (50.0)	7 (53.8)
Treatment-related	1 (33.3)	1 (33.3)	0	2 (22.2)	2 (28.6)	1 (16.7)	3 (23.1)
TEAE leading to treatment discontinuation	1 (33.3)	0	0	1 (11.1)	1 (14.3)	1 (16.7)	2 (15.4)
Treatment-related	1 (33.3)	0	0	1 (11.1)	1 (14.3)	0	1 (7.7)
TEAEs leading to death	0	0	0	0	1 (14.3)	0	1 (7.7)
Treatment-related	0	0	0	0	0	0	0
Most common treatment-related TEAEs (grade ≥ 3)							
Decreased appetite	0	0	1 (33.3)	1 (11.1)	0	0	0
Fulminant type 1 diabetes mellitus	0	1 (33.3)	0	1 (11.1)	0	0	0
Hyperuricemia	0	1 (33.3)	0	1 (11.1)	0	0	0
Hyperglycemia	0	0	0	0	1 (14.3)	0	1 (7.7)
Abnormal hepatic function	1 (33.3)	0	0	1 (11.1)	0	0	0
Increased aspartate aminotransferase	0	1 (33.3)	0	1 (11.1)	1 (14.3)	0	1 (7.7)
Increased blood alkaline phosphatase	0	1 (33.3)	0	1 (11.1)	0	1 (16.7)	1 (7.7)
Increased gamma-glutamyl transferase	0	1 (33.3)	0	1 (11.1)	0	0	0
Increased lipase	0	0	0	0	0	1 (16.7)	1 (7.7)
Increased alanine aminotransferase	0	0	0	0	1 (14.3)	0	1 (7.7)
Anemia	0	0	0	0	1 (14.3)	1 (16.7)	2 (15.4)
Lymphopenia	0	0	0	0	1 (14.3)	0	1 (7.7)
Tumor pain	0	0	0	0	0	1 (16.7)	1 (7.7)
Bronchial obstruction	0	0	0	0	0	1 (16.7)	1 (7.7)
Hiatus hernia	0	0	0	0	1 (14.3)	0	1 (7.7)
Suicide	0	0	0	0	1 (14.3)	0	1 (7.7)

AE, adverse event; Q2W, every 2 weeks; Q4W, every 4 weeks; QD, once daily; TEAE, treatment-emergent adverse event

In phase 1b part, all patients (13/13 [100.0%]) experienced ≥ 1 TEAEs (Table 2). The most common TEAEs of grade ≥ 3 include anemia (2/13 [15.4%] patients), hyperglycemia, lymphopenia, tumor pain, bronchial obstruction, hiatus hernia, suicide, increased aspartate aminotransferase, blood alkaline phosphatase, lipase, alanine aminotransferase (1/13 [7.7%] patients each). Serious TEAEs were reported in 5/13 (38.5%) patients (cetrelimab 240 mg Q2W + erdafitinib 6 mg QD: 3/7 [42.9%] patients; cetrelimab 240 mg Q2W + erdafitinib 8 mg QD: 2/6 [33.3%] patients) and among them 2/13 (15.4%) patients had treatment-related serious TEAEs. Hiatus hernia, tumor pain, completed suicide, bronchial obstruction, and Stevens-Johnson syndrome (1/13 [7.7%] patients each) were the reported serious TEAEs of grade ≥ 3. Treatment-emergent irAEs were observed in 2/13 (15.4%) patients in cetrelimab 240 mg Q2W + erdafitinib 6 mg QD cohort, but none in cetrelimab 240 mg Q2W + erdafitinib 8 mg QD cohort. Overall, 3 death cases were reported in phase 1b part (1 patient due to suicide in cetrelimab 240 mg + erdafitinib 6 mg cohort; 2 patients in erdafitinib 8 mg cohort due to PD). Cetrelimab-related irAEs of special interest were reported in 2/13 (15.4%) patients, respectively (Online Resource 1). Hyperphosphatemia, the most common AE associated with FGFR inhibitors was reported in 11/13 (84.6%) patients. Infusion-related reactions and treatment-related deaths were not reported in phase 1b part. No clinically meaningful changes in vital signs, ECG, ECOG performance status, and infusion-related events were observed in phase 1a and 1b parts of the study.

#### DLTs

No DLTs were observed in all three cohorts of phase 1a part. Whereas in phase 1b part, 1/13 (7.7%) patients who was on cetrelimab 240 mg Q2W + erdafitinib 6 mg QD combination therapy reported Stevens-Johnson syndrome on day 26, which was considered as grade 3 serious irAE by the investigator which led to dose interruption. This grade 3 AE was improved to grade 2 on day 40. Based on safety data, the dosing regimen of 480 mg Q4W was chosen as the RP2D of cetrelimab monotherapy, and 240 mg Q2W was selected as the recommended dose for conducting phase 1b part of this study. For combination therapy, cetrelimab 240 mg Q2W + erdafitinib 8 mg QD was determined as RP2D.

### Pharmacokinetics

After a single intravenous infusion of cetrelimab, mean maximum serum concentration (C_max_) and area under the concentration-time curve to 2 week (AUC_2wk_) were 27.5 µg/mL and 4576 µg·h/mL for cetrelimab 80 mg, and 83.8 µg/mL and 13,578 µg·h/mL for cetrelimab 240 mg. Systemic exposure (C_max_, AUC_2wk_) has been increased 3-fold with the 3-fold increase in dosing level. C_max_ of 240 mg cetrelimab given along with erdafitinib 6 mg and 8 mg was comparable (82.4 µg/mL and 89.6 µg/mL, respectively). Results of the 480 mg cohort were not added as they were limited observations. Mean plasma total erdafitinib concentrations, when given with cetrelimab were higher for the 8 mg QD dose. Cetrelimab (240 mg) when administered with erdafitinib was rapidly absorbed, with a median T_max_ of 2 to 3 h. The median T_max_, mean C_max_, AUC_2wk_, and C_trough_ at cycle 1 day 15 were comparable between the single intravenous infusion of cetrelimab (240 mg) alone and combined with erdafitinib (Table 3).

**Table 3:** PK parameters of cetrelimab (PK analysis set)

Parameters	Cetrelimab			
	80 mg Q2W	240 mg Q2W	240 mg Q2W + erdafitinib 6 mg QD	240 mg Q2W + erdafitinib 8 mg QD
Cycle 1 day 1				
n	3	3	7	6
C_max_ (µg/mL)	27.5 (3.45)	83.8 (17.6)	82.4 (24.2)	89.6 (17.8)
T_max_ (h), median (range)	3.03 (3.03–3.13)	1.08 (1.00–1.18)	1.02 (0.95–6.55)	1.11 (0.97–1.58)
AUC_2wk_ (µg·h/mL)	4576 (471)	13,578 (3460)	13,147 (2820)	12,611 (3198)
Cycle 1 day 15				
n	3	3	7	6
C_trough_ (µg/mL)	8.30 (1.66)	22.5 (1.04)	24.4 (5.36)	25.1 (4.50)
Cycle 2 day 1				
n	3	-	6	6
C_trough_ (µg/mL)	11.8 (2.83)	-	43.1 (8.61)	39.5 (7.06)
Cycle 3 day 1				
n	-	-	4	3
C_trough_ (µg/mL)	-	-	65.1 (17.4)	63.3 (16.9)
Cycle 4 day 1				
n	-	-	3	-
C_trough_ (µg/mL)	-	-	62.4 (16.5)	-
C_max_ (µg/mL)	-	-	141 (8.40)	-
T_max_ (h)	-	-	2.37 (0.55–2.58)	-
t_1/2_ (h)	-	-	382.4 (204.2)	-
CL (l/h)	-	-	0.00810 (0.00264)	-

All values are expressed in terms of mean (SD) unless specified. AUC_2wk_, area under the concentration-time curve from 0 to 2 week; C_max_, maximum serum concentration; C_trough_, serum concentration just prior to next administration of drug; CL, total systemic clearance; PK, pharmacokinetics; Q2W, every 2 weeks; Q4W, every 4 weeks; QD, once daily; SD, standard deviation; t_1/2_, half-life; T_max_, time to maximum concentration

**Fig. 1:**
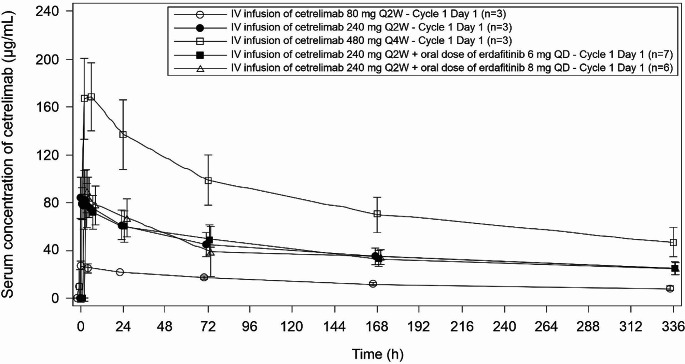
Serum concentration (mean [SD]) versus time profiles of cetrelimab alone and combination therapy at all dosing levels (PK analysis set; cycle 1 day 1) IV, intravenous; PK, pharmacokinetics; Q2W, every 2 weeks; Q4W, every 4 weeks; QD, once daily; SD, standard deviation

PK parameters of erdafitinib are presented in Online Resource 2. A linear and dose-related rise in the exposure to cetrelimab was observed when given as single agent. The exposure to cetrelimab was found unaffected when combined with erdafitinib (Fig. 1).

### Immunogenicity

No patient in phase 1a part was anti-cetrelimab antibody positive, and 1/6 (16.7%) patient in the phase 1b part (cetrelimab 240 mg + erdafitinib 8 mg cohort) was positive for anti-cetrelimab antibodies with a titer of 6400 at baseline, but no treatment-induced anti-cetrelimab antibodies were detected.

### Pharmacodynamics

PD-1 receptor occupancy by cetrelimab on circulating CD3+, CD3+/CD4+, and CD3+/CD8 + cells, measured by flow cytometry reached 100% within 2 h post-dose and remained saturated throughout all doses in both phase 1a and 1b parts of the study. Similar receptor occupancy was observed at both RP2Ds (cetrelimab 480 mg Q4W and cetrelimab 240 mg Q2W + erdafitinib 8 mg QD).

### Efficacy

In phase 1a part of the study, 9/9 (100%) patients were evaluated for efficacy. ORR (complete response [CR] + partial response [PR]) was 11.1% (1/9 patients) and 1/3 (33.3%) patients in cetrelimab 480 mg cohort had confirmed partial response (PR). Overall median DOR was 16.82 months (95% confidence interval [CI]: not evaluable [NE]–NE) and PFS was 8.31 months (95% CI: 0.95–NE). Disease control rate (DCR; CR + PR + stable disease [SD] assessed for ≥ 16 weeks) was 44.4% (4/9 patients) (Online Resource 3). Tumor shrinkage was observed in 4/8 patients (50% had > 30% tumor shrinkage) and 1 patient did not have target lesion (Online Resource 4).

In phase 1b part of the study, 12/13 (92.3%) patients were evaluated for efficacy. The ORR (CR + PR) was 16.7% (2/12 patients). In the cetrelimab 240 mg + erdafitinib 6 mg cohort, none had confirmed PR, whereas in cetrelimab 240 mg + erdafitinib 8 mg cohort 2/6 (33.3%) patients had confirmed PR. Overall median DOR was 2.73 months (range: NE–NE) and PFS was 2.76 months (95% CI: 1.31–4.14). In 2 patients with *FGFR* gene aberration, 1 patient reported confirmed PR and PFS was 4.14 months and 2.76 months, respectively. DCR (CR + PR + SD assessed for ≥ 16 weeks) was 25.0% (3/12 patients) in patients on combination therapy (Online Resource 3). Of 12 patients, 7 had tumor reduction, and among them, 3 patients had > 30% tumor reduction and 1 patient did not have target lesion (Online Resource 4).

## Discussion

Target-specific therapies including immune-targeted and pathway-targeted drugs contribute to the success of current cancer care modalities and overall cancer management [18]. In this dose-escalation phase 1/1b study, cetrelimab as a single agent and in combination with erdafitinib was assessed for DLTs, safety, PK, immunogenicity, pharmacodynamics, and antitumor activity in Japanese patients with advanced solid tumors. Cetrelimab ± erdafitinib had an acceptable safety profile at all dose levels. No DLTs were reported in patients on monotherapy. The only DLT observed in 1 patient who was on combination therapy was Stevens-Johnson syndrome (grade 3), which was considered to be immune-related, and probably related to cetrelimab by the investigator. Immuno checkpoint inhibitors are known to be associated with a rare but serious skin AE, Stevens-Johnson syndrome [19]. Upon comparison of safety profiles, the RP2Ds were determined as cetrelimab 480 mg Q4W for monotherapy and cetrelimab 240 mg Q2W + erdafitinib 8 mg QD for combination therapy. Safety profile of cetrelimab observed in this study of Japanese population was consistent with those observed in the global study [8], and the RP2Ds determined are the same as global RP2Ds [8, 20].

Safety findings suggest that cetrelimab as a single agent was acceptable at all dosing levels and the safety profile was consistent with the global population assessing cetrelimab [8] as well as with other PD-1 inhibitors, although the sample size is limited [21–25]. Compared to the global study, a lower proportion of patients on monotherapy reported ≥ 1 TEAE [8]. Grade ≥ 3 TEAEs were rare and most of them were grade 1 and 2. Although AEs associated with erdafitinib were observed in the combination part of the study, no new safety signals were observed [13, 14, 17]. Overall safety profile of the combination therapy is consistent with cetrelimab and erdafitinib monotherapies in global and Japanese population studies [8, 13, 14, 17].

A dose-dependent increase in cetrelimab exposure (C_max_, AUC_2wk_) in phase 1a part was observed with doses ranging between 80 mg and 240 mg. In phase 1b part, the exposure of cetrelimab was found unaffected by the addition of erdafitinib. The C_trough_ at cycle 1 day 15 of cetrelimab 240 mg alone and cetrelimab 240 mg + erdafitinib were comparable. Higher exposures were observed at day 1 of subsequent cycles compared to cycle 1 day 1. The clearance of cetrelimab at 240 mg dose (8.1 mL/h) was comparable with the global study (8.6 mL/h) and other PD-1 blockers, nivolumab (9.5 mL/h) and pembrolizumab (9.2 mL/h) [8, 26, 27]. PK results of cetrelimab were unaffected when given along with erdafitinib in combination groups collectively implying that there are no significant drug interactions although a slight degree of variability was observed. Overall, the PK findings of cetrelimab in Japanese patients were consistent with the global population [8]. Anti-cetrelimab antibodies were detected only in one patient who was on combination therapy, but they didn’t appear to have an impact on the PKs of cetrelimab inferring that anti-cetrelimab antibodies have no impact on clinical activity. No treatment-induced anti-cetrelimab antibodies were present as observed in the global study assessing cetrelimab and the studies of other PD-1 blockers [8, 21, 24]. PD-1 receptor occupancy of cetrelimab was found to be 100% within 2 h post-dose at all dosing levels suggesting that cetrelimab is binding selectively to PD-1 receptor thus effectively targeting the cancer cells.

Despite the absence of a proven complete response during efficacy analysis, 1 patient receiving cetrelimab 480 mg and 2 patients on cetrelimab 240 mg and erdafitinib 8 mg had confirmed PR. DCR (CR + PR + SD assessed for ≥ 16 weeks) was observed in almost half of the patients on monotherapy and one-fourth of the patients on combination therapy. PR was observed in 3 patients (1 patient on cetrelimab 480 mg and 2 patients on cetrelimab 240 mg and erdafitinib 8 mg). Taken together, the efficacy findings of cetrelimab in patients with solid tumors demonstrated preliminary antitumor activity which may support further study of cetrelimab as a mono and combination therapy in Japan. We acknowledge some inherent limitations, including small sample size from only two sites which restricts the generalizability of the findings, and the lack of emphasis on specific types of tumors in the study that hindered the scope of understanding the activity of the study drug on different tumors.

In conclusion, the RP2Ds of cetrelimab in the Japanese population were established in this dose-escalation phase 1/1b study and these doses are same as those established in the global study. Cetrelimab demonstrated manageable safety and preliminary antitumor activity both as monotherapy and in combination with erdafitinib at all dose levels. Overall, the findings of this study support the inclusion of Japanese population in future global oncology studies that assess cetrelimab with/without erdafitinib.

### Electronic supplementary material

Below is the link to the electronic supplementary material.

Supplementary Material 1

Supplementary Material 2

Supplementary Material 3

Supplementary Material 4

## Data Availability

These data are out of scope for our data sharing policy.
