# Supplementary material for: A phase 1/1b, open-label, dose-escalation study of PD-1 inhibitor, cetrelimab alone and in combination with FGFR inhibitor, erdafitinib in Japanese patients with advanced solid tumors
Source: Invest New Drugs. 2024 Jun 4;42(4):376–85. doi: 10.1007/s10637-024-01433-3 (PMC11327176; doi:10.1007/s10637-024-01433-3)
Supplement: Supplementary file 1 — Supplementary Material 1 [file 10637_2024_1433_MOESM1_ESM.pdf]

SUPPLEMENTARY MATERIAL

A phase 1/1b, open-label, dose-escalation study of PD-1 inhibitor, cetrelimab alone and in combination with FGFR inhibitor, erdafitinib in Japanese patients with advanced solid tumors

Journal name: Investigational New Drugs

Noboru Yamamoto[1], Yasutoshi Kuboki[2], Kenichi Harano[2], Takafumi Koyama[1], Shunsuke Kondo[1], Akiko Hagiwara[3], Noriko Suzuki[3], Ei Fujikawa[3], Kiichiro Toyoizumi[3], Mayumi Mukai[3], Toshihiko Doi[2*]

[1]Department of Experimental Therapeutics, National Cancer Center Hospital, Tokyo, Japan, [2]Department of Experimental Therapeutics, National Cancer Center Hospital East, Chiba, Japan, [3]Research and Development Division, Janssen Pharmaceutical K.K., Tokyo, Japan.

***Corresponding author**:

Dr. Toshihiko Doi

Department of Experimental Therapeutics

National Cancer Center Hospital East, Chiba, Japan

Phone no: +81-4-7133-1111

Email: tdoi@east.ncc.go.jp

Table S1 TEAEs of special interest (all-treated analysis set)

a) Cetrelimab-related

**Characteristics, n (%)**	**Phase 1a**				**Phase 1b**		
	80 mg Q2W (n=3)	240 mg Q2W (n=3)	480 mg Q4W (n=3)	Total (n=9)	Cetrelimab 240 mg Q2W + erdafitinib 6 mg QD (n=7)	Cetrelimab 240 mg Q2W + erdafitinib 8 mg QD (n=6)	Total (n=13)
Patients with ≥1 TEAE of special interest	2 (66.7)	2 (66.7)	0	4 (44.4)	2 (28.6)	0	2 (15.4)
Treatment-emergent irAEs	2 (66.7)	2 (66.7)	0	4 (44.4)	2 (28.6)	0	2 (15.4)
Rash	1 (33.3)	2 (66.7)	0	3 (33.3)	0	0	0
Pruritus	1 (33.3)	0	0	1 (11.1)	0	0	0
Stevens-Johnson syndrome	0	0	0	0	1 (14.3)	0	1 (7.7)
Adrenal insufficiency	0	1 (33.3)	0	1 (11.1)	0	0	0
Hyperthyroidism	1 (33.3)	0	0	1 (11.1)	1 (14.3)	0	1 (7.7)
Hypothyroidism	0	0	0	0	1 (14.3)	0	1 (7.7)
Abnormal hepatic function	1 (33.3)	0	0	1 (11.1)	0	0	0
Fulminant type 1 diabetes mellitus	0	1 (33.3)	0	1 (11.1)	0	0	0
Myalgia	1 (33.3)	0	0	1 (11.1)	0	0	0

Q2W, every 2 weeks; Q4W, every 4 weeks; QD, once daily; irAE, immune-related adverse event; TEAE, treatment-emergent adverse event

b) Erdafitinib-related

**Characteristics, n (%)**	**Phase 1b**		
	Cetrelimab 240 mg Q2W + erdafitinib 6 mg QD (n=7)	Cetrelimab 240 mg Q2W + erdafitinib 8 mg QD (n=6)	Total (n=13)
Patients with ≥1 TEAE of special interest	7 (100.0)	6 (100.0)	13 (100.0)
TEAEs of special interest			
Hyperphosphatemia	5 (71.4)	6 (100.0)	11 (84.6)
Paronychia	0	2 (33.3)	2 (15.4)
Nail discoloration	2 (28.6)	1 (16.7)	3 (23.1)

Nail disorder	0	1 (16.7)	1 (7.7)
Onycholysis	0	1 (16.7)	1 (7.7)
Stomatitis	3 (42.9)	2 (33.3)	5 (38.5)
Dry mouth	1 (14.3)	0	1 (7.7)
Dry skin	0	2 (33.3)	2 (15.4)
Retinopathy	1 (14.3)	0	1 (7.7)

Q2W, every 2 weeks; Q4W, every 4 weeks; QD, once daily; TEAE, treatment-emergent adverse event.
