# Supplementary material for: A phase 1/1b, open-label, dose-escalation study of PD-1 inhibitor, cetrelimab alone and in combination with FGFR inhibitor, erdafitinib in Japanese patients with advanced solid tumors
Source: Invest New Drugs. 2024 Jun 4;42(4):376–85. doi: 10.1007/s10637-024-01433-3 (PMC11327176; doi:10.1007/s10637-024-01433-3)
Supplement: Supplementary file 2 — Supplementary Material 2 [file 10637_2024_1433_MOESM2_ESM.pdf]

SUPPLEMENTARY MATERIAL

A phase 1/1b, open-label, dose-escalation study of PD-1 inhibitor, cetrelimab alone and in combination with FGFR inhibitor, erdafitinib in Japanese patients with advanced solid tumors

Journal name: Investigational New Drugs

Noboru Yamamoto[1], Yasutoshi Kuboki[2], Kenichi Harano[2], Takafumi Koyama[1], Shunsuke Kondo[1], Akiko Hagiwara[3], Noriko Suzuki[3], Ei Fujikawa[3], Kiichiro Toyoizumi[3], Mayumi Mukai[3], Toshihiko Doi[2*]

[1]Department of Experimental Therapeutics, National Cancer Center Hospital, Tokyo, Japan,

[2]Department of Experimental Therapeutics, National Cancer Center Hospital East, Chiba, Japan,

[3]Research and Development Division, Janssen Pharmaceutical K.K., Tokyo, Japan.

***Corresponding author**:

Dr. Toshihiko Doi

Department of Experimental Therapeutics

National Cancer Center Hospital East, Chiba, Japan

Phone no: +81-4-7133-1111

Email: tdoi@east.ncc.go.jp

Table S2 PK parameters of erdafitinib (PK analysis set)

Parameters	Cetrelimab 240 mg Q2W + erdafitinib 6 mg QD	Cetrelimab 240 mg Q2W + erdafitinib 8 mg QD
Cycle 1 day 1		
N	7	6
C_{trough} (ng/mL)	BQL	BQL
C_{max} (ng/mL)	290 (79.5)	474 (257.0)
Cycle 1 day 2		
N	7	6
C_{trough} (ng/mL)	204 (76.8)	427 (245.0)
Cycle 1 day 15		
N	7	6
C_{trough} (ng/mL)	833 (401.0)	2334 (1656.0)
Cycle 2 day 1		
N	4	5
C_{trough} (ng/mL)	710 (374.0)	2403 (1770.0)
Cycle 3 day 1		
N	4	-
C_{trough} (ng/mL)	545 (239.0)	-
Cycle 4 day 1		
N	3	-
C_{trough} (ng/mL)	566 (319.0)	-
C_{max} (ng/mL)	750 (386.0)	-

All values are expressed in terms of mean (SD). BQL, below quantification limit; C_{max}, maximum serum concentration; C_{trough}, serum concentration just prior to next administration of drug; Q2W, every 2 weeks; Q4W, every 4 weeks; QD, once daily, SD, standard deviation.
