# Supplementary material for: A phase 1/1b, open-label, dose-escalation study of PD-1 inhibitor, cetrelimab alone and in combination with FGFR inhibitor, erdafitinib in Japanese patients with advanced solid tumors
Source: Invest New Drugs. 2024 Jun 4;42(4):376–85. doi: 10.1007/s10637-024-01433-3 (PMC11327176; doi:10.1007/s10637-024-01433-3)
Supplement: Supplementary file 3 — Supplementary Material 3 [file 10637_2024_1433_MOESM3_ESM.pdf]

SUPPLEMENTARY MATERIAL

A phase 1/1b, open-label, dose-escalation study of PD-1 inhibitor, cetrelimab alone and in combination with FGFR inhibitor, erdafitinib in Japanese patients with advanced solid tumors

Journal name: Investigational New Drugs

Noboru Yamamoto[1], Yasutoshi Kuboki[2], Kenichi Harano[2], Takafumi Koyama[1], Shunsuke Kondo[1], Akiko Hagiwara[3], Noriko Suzuki[3], Ei Fujikawa[3], Kiichiro Toyoizumi[3], Mayumi Mukai[3], Toshihiko Doi[2*]

[1]Department of Experimental Therapeutics, National Cancer Center Hospital, Tokyo, Japan,

[2]Department of Experimental Therapeutics, National Cancer Center Hospital East, Chiba, Japan,

[3]Research and Development Division, Janssen Pharmaceutical K.K., Tokyo, Japan.

***Corresponding author**:

Dr. Toshihiko Doi

Department of Experimental Therapeutics

National Cancer Center Hospital East, Chiba, Japan

Phone no: +81-4-7133-1111

Email: tdoi@east.ncc.go.jp

Table S3 Summary of efficacy results (response evaluable analysis set)

Parameters	Phase 1a (cetrelimab)				Phase 1b (cetrelimab + erdafitinib)		
	80 mg Q2W (n=3)	240 mg Q2W (n=3)	480 mg Q4W (n=3)	Total (n=9)	240 mg Q2W + 6 mg QD (n=6)	240 mg Q2W + 8 mg QD (n=6)	Total (n=12)
BOR							
CR	0	0	0	0	0	0	0
PR	0	0	1 (33.3)	1 (11.1)	0	2 (33.3)	2 (16.7)
SD[a]	0	2 (66.7)	1 (33.3)	3 (33.3)	2 (33.3)	0	2 (16.7)
PD	2 (66.7)	1 (33.3)	0	3 (33.3)	4 (66.7)	4 (66.7)	8 (66.7)
Neither CR nor PD	1 (33.3)	0	0	1 (11.1)	0	0	0
NE	0	0	1 (33.3)	1 (11.1)	0	0	0
ORR (CR+PR)	0	0	1 (33.3)	1 (11.1)	0	2 (33.3)	2 (16.7)
95% CI	0.0–70.8	0.0–70.8	0.8–90.6	0.3–48.2	0.0–45.9	4.3–77.7	2.1–48.4
DCR (CR+PR+SD[a])	0	2 (66.7)	2 (66.7)	4 (44.4)	1 (16.7)	2 (33.3)	3 (25.0)
95% CI	0.0–70.8	9.4–99.2	9.4–99.2	13.7–78.8	0.4–64.1	4.3–77.7	5.5–57.2
PFS duration (months)[b]							
25th percentile (95% CI)	1.87 (1.87–NE)	0.95 (0.95– NE)	0.99 (0.99–NE)	1.87 (0.95–8.31)	1.31 (1.18–2.89)	1.38 (1.25–2.76)	1.38 (1.18–2.76)
Median (95% CI)	1.87 (1.87–NE)	11.30 (0.95–NE)	8.31 (0.99–NE)	8.31 (0.95– NE)	2.89 (1.18–5.29)	2.10 (1.25–NE)	2.76 (1.31–4.14)
75th percentile (95% CI)	NE (1.87–NE)	13.80 (0.95–NE)	22.18 (0.99–NE)	13.80 (1.87–NE)	5.29 (2.56–NE)	4.14 (1.38–NE)	4.14 (2.56–NE)

All values are expressed in terms of n (%). [a]SD assessed for at least 16 weeks. [b]Based on KM estimate. BOR, best overall response; CI, confidence interval; CR, complete response; DCR, disease control rate; KM, Kaplan-Meier; NE, not evaluable; ORR, overall response rate; PD, progressive disease; PFS, progression-free survival; PR, partial response; Q2W, every 2 weeks; Q4W, every 4 weeks; QD, once daily; SD, stable disease.
