# Supplementary material for: A phase 1/1b, open-label, dose-escalation study of PD-1 inhibitor, cetrelimab alone and in combination with FGFR inhibitor, erdafitinib in Japanese patients with advanced solid tumors
Source: Invest New Drugs. 2024 Jun 4;42(4):376–85. doi: 10.1007/s10637-024-01433-3 (PMC11327176; doi:10.1007/s10637-024-01433-3)
Supplement: Supplementary file 4 — Supplementary Material 4 [file 10637_2024_1433_MOESM4_ESM.pdf]

SUPPLEMENTARY MATERIAL

A phase 1/1b, open-label, dose-escalation study of PD-1 inhibitor, cetrelimab alone and in combination with FGFR inhibitor, erdafitinib in Japanese patients with advanced solid tumors

Journal name: Investigational New Drugs

Noboru Yamamoto[1], Yasutoshi Kuboki[2], Kenichi Harano[2], Takafumi Koyama[1], Shunsuke Kondo[1], Akiko Hagiwara[3], Noriko Suzuki[3], Ei Fujikawa[3], Kiichiro Toyoizumi[3], Mayumi Mukai[3], Toshihiko Doi[2*]

[1]Department of Experimental Therapeutics, National Cancer Center Hospital, Tokyo, Japan,

[2]Department of Experimental Therapeutics, National Cancer Center Hospital East, Chiba, Japan,

[3]Research and Development Division, Janssen Pharmaceutical K.K., Tokyo, Japan.

***Corresponding author**:

Dr. Toshihiko Doi

Department of Experimental Therapeutics

National Cancer Center Hospital East, Chiba, Japan

Phone no: +81-4-7133-1111

Email: tdoi@east.ncc.go.jp

Fig. S1: Waterfall plot of percentage change in tumor size for each patient

a) Phase 1a

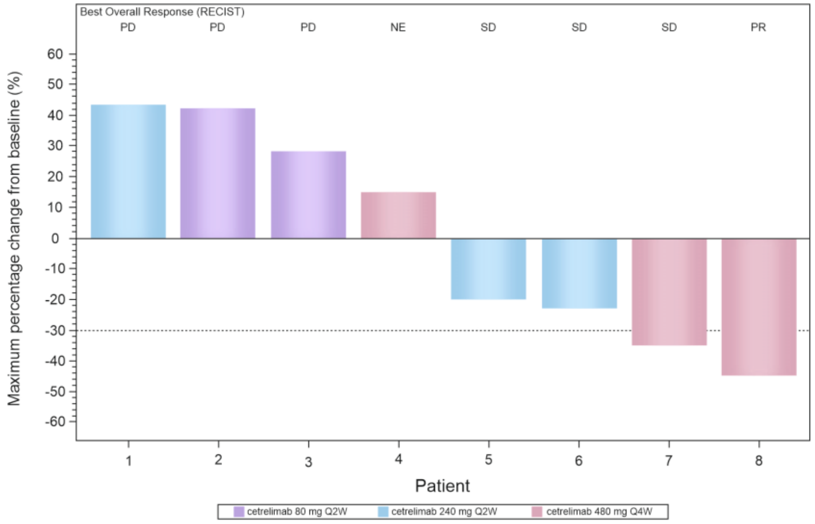

b) Phase 1b

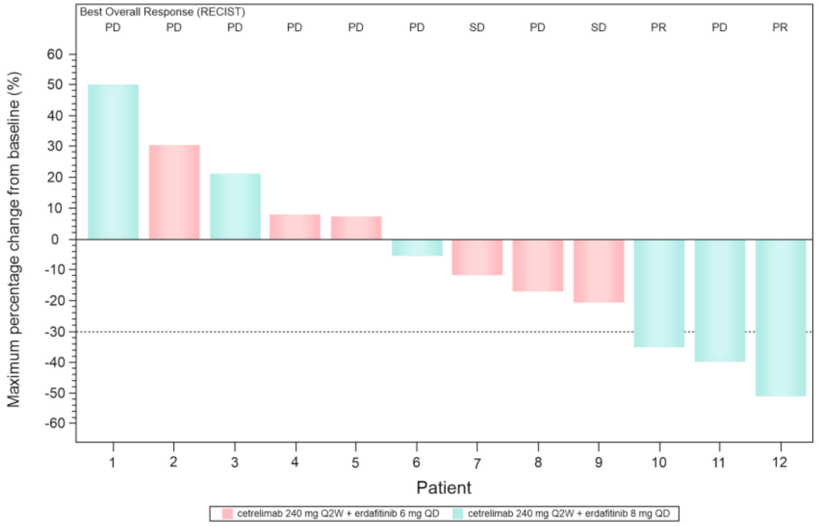

Note: One patient in phase 1a part does not have target lesion. NE, not evaluable; PD, progressive disease, PR, partial response, Q2W, every 2 weeks; Q4W, every 4 weeks; QD, once daily; RECIST, response evaluation criteria in solid tumors; SD, stable disease.
